# Exercise Improves the Cytoskeletal and Metabolic Functions of Brown Adipocytes Through the ADRβ3/COX2-Ywhah Axis

**DOI:** 10.3390/ijms26072978

**Published:** 2025-03-25

**Authors:** Jingzhe Xiao, Chunyan Xu, Rongxin Zhu, Pengyu Fu, Jie Jia, Lijing Gong

**Affiliations:** 1 China Ice Sport College, Beijing Sport University, Beijing 100084, China; xiaojingzhe1120@bsu.edu.cn; 2 Key Laboratory of Physical Fitness and Exercise, Ministry of Education, Beijing Sport University, Beijing 100084, China; 3 School of Sport Science, Beijing Sport University, Beijing 100084, China; 4 Shanghai Research Institute of Sports Science, Shanghai 200030, China; zhurongxin@shriss.cn; 5 Department of Physical Education, Northwestern Polytechnical University, Xi’an 710072, China; fupy@nwpu.edu.cn; 6 Beijing Research Institute of Sports Science, Beijing 100075, China; jiajie@tyj.beijing.gov.cn

**Keywords:** obesity, brown adipose tissue, metabolism, Ywhah, 14-3-3η

## Abstract

Brown adipose tissue (BAT) is a critical target for obesity treatment, and exercise can enhance BAT function through the activation of ADRβ3. However, the molecular mechanisms underlying BAT metabolism following the exercise-induced activation of ADRβ3 remain unclear. This study utilized RNA sequencing, Western blotting, Oil Red O staining, weighted gene co-expression network analysis (WGCNA), and machine learning to investigate the role of the ADRβ3-COX2 pathway in lipid metabolism in brown adipocytes. We identified Ywhah as a key gene and validated our findings using external datasets. Our results demonstrate that exercise significantly enhances brown adipose tissue metabolism in mice, with ADRβ3 activation promoting metabolic activity in brown adipocytes. In contrast, COX2 inhibition notably reduced the lipolytic effect and thermogenic gene expression induced by ADRβ3 activation. WGCNA and machine learning identified Ywhah as the most important feature variable in the downstream signaling of the ADRβ3-COX2 pathway. External microarray data further confirmed that 8 weeks of aerobic exercise significantly upregulated Ywhah expression. Additionally, Ywhah displayed strong binding affinity to cytoskeletal proteins in affinity purification–mass spectrometry experiments, and its expression was highly correlated with cytoskeletal GSVA scores. In summary, this study reveals the potential role of the ADRβ3-COX2-Ywhah-cytoskeleton axis in regulating brown adipocyte metabolism, providing new insights into obesity treatment mechanisms.

## 1. Introduction

Obesity has become a major global public health issue, necessitating effective intervention strategies. Brown adipose tissue (BAT) has gained considerable attention in recent years as a potential target for anti-obesity therapies [1]. BAT not only plays a key role in non-shivering thermogenesis but also is involved in the regulation of glucose and lipid metabolism [2,3]. Studies have shown that BAT transplantation can improve glucose tolerance, enhance insulin sensitivity, and reduce fat accumulation in recipient mice [4]. Although the effect of exercise on BAT thermogenesis remains controversial [5,6], substantial evidence indicates that exercise promotes BAT metabolic function, with the sympathetic nervous system and its adrenergic signaling pathways playing a crucial role [7].

It is worth noting that our previous research, through RNA microarray sequencing, found significant changes in genes related to the ADRβ3-COX2 pathway in the BAT of obese mice following aerobic exercise [8]. Adrenergic receptors (ADRs) are divided into α and β subtypes, with the β subtype further categorized into β1, β2, and β3 [9]. ADRβ3 is primarily expressed in adipocytes, with particularly high abundance in BAT [10]. Isoproterenol (ISO) is a non-selective ADRβ agonist that activates all β subtypes [11]. Given the involvement of fluid regulation and sympathetic nervous system activation during exercise, this study examines BAT in obese mice after aerobic exercise intervention and uses ISO to simulate sympathetic activation at the cellular level. The aim is to explore exercise-induced stimulation and its role in the metabolic regulation of brown adipocytes.

Cyclooxygenase-2 (COX2) is a key rate-limiting enzyme in prostaglandin synthesis, catalyzing the conversion of arachidonic acid into various prostaglandins [12]. The activation of the COX2/prostaglandin pathway significantly enhances the activity of BAT [13]. Furthermore, studies have shown that exercise increases COX2 expression in both skeletal muscle and white adipose tissue (WAT) [14], and elevated COX2 levels promote the browning of WAT [15]. However, the cold-induced browning of WAT appears to be independent of COX2 [16], suggesting that COX2 may play a specific role in exercise-induced changes in adipose tissue function. Therefore, ISO may enhance BAT function by regulating COX2, but the exact relationship between ADRβ3 and COX2 in BAT remains unclear. In our previous study of obese mice, we found that the ADRβ3/VEGF/COX2 axis plays a key role in the exercise-induced improvement of BAT function [8], but the causal relationship and underlying molecular mechanisms require further investigation.

The protein encoded by Ywhah, 14-3-3η, is a highly conserved molecule in eukaryotic cells, primarily regulating the function of its target proteins by binding to their phosphorylated sites [17]. However, the specific role of 14-3-3η in energy metabolism has not been fully studied. Evidence suggests that the deletion of 14-3-3η exacerbates high-fat diet-induced diabetic cardiomyopathy by disrupting myocardial fatty acid metabolism [18]. Nevertheless, the exact function of the 14-3-3 protein family, particularly 14-3-3η, in BAT remains unclear. Additionally, it has yet to be confirmed whether the 14-3-3 family is regulated by exercise. Cytoskeletal changes play an important role in BAT development. During adipogenesis, cytoskeletal rearrangement precedes nucleolus remodeling, and as adipocytes differentiate and mature, their cytoskeleton gradually becomes more relaxed [19]. A more relaxed cytoskeleton also facilitates the development of thermogenic programs in BAT [20]. However, whether exercise accelerates the cytoskeletal rearrangement process and the underlying mechanisms remain unknown.

In summary, we propose the hypothesis that exercise enhances Ywhah (14-3-3η) expression through the activation of the ADRβ3/COX2 pathway, thereby promoting cytoskeletal remodeling and metabolic function in BAT. To validate this hypothesis, we used ISO to activate ADRβ3 in brown adipocytes and/or inhibit COX2, dynamically monitoring changes in lipid metabolism. Additionally, key feature genes were identified through weighted gene co-expression network analysis (WGCNA) and machine learning methods, with further validation using external microarray datasets. This study provides new insights into the regulatory mechanisms between exercise and BAT function, laying a theoretical foundation for the development of novel anti-obesity strategies.

## 2. Results

### 2.1. Exercise Enhances Lipid Metabolic Function in Brown Adipose Tissue

**Figure 1:**
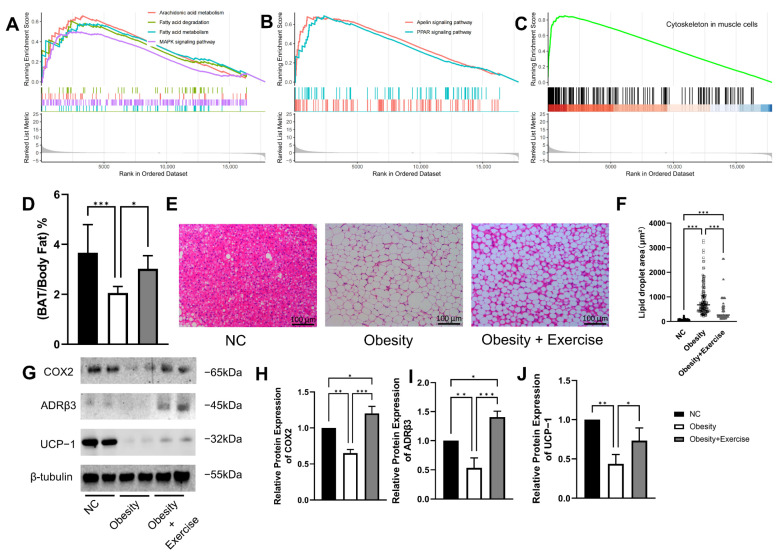
Effects of exercise on brown adipose tissue. (**A**–**C**) GSEA of transcriptomic data after aerobic exercise. (**D**) Changes in the proportion of brown adipose tissue in total body fat after 8 weeks of aerobic exercise. (**E**,**F**) H&E staining shows that exercise alleviates obesity-induced lipid droplet accumulation and significantly reduces droplet size. Scale bar: 100 μm. (**G**–**J**) Western blot results confirm that exercise restores the expression of COX2, a key enzyme in the arachidonic acid metabolism pathway, and increases the levels of ADRβ3 and UCP-1. NC: normal diet control, each n = 8. * *p* < 0.05, ** *p* < 0.01, *** *p* < 0.001.

To investigate how exercise affects BAT, we first analyzed data from GSE207705 [21]. The results revealed that after 8 weeks of aerobic exercise, pathways related to lipid metabolism in BAT were significantly upregulated, with the arachidonic acid metabolism pathway showing the most prominent activation (Figure 1A). In addition, the Apelin pathway, associated with lipid transport, and the PPAR pathway, which plays a key role in insulin sensitivity, were also significantly enhanced (Figure 1B). Notably, cytoskeleton-related pathways were also significantly upregulated (Figure 1C).

Next, we validated the bioinformatics analysis results. After 8 weeks of aerobic exercise, overall fat mass was significantly reduced; however, unlike WAT, the proportion of BAT in obese mice did not decrease but rather increased (Figure 1D). Hematoxylin and eosin (H&E) staining results showed that obesity led to excessive lipid droplet accumulation in BAT, while exercise intervention significantly alleviated this phenomenon and notably reduced the volume of lipid droplets (Figure 1E,F).

Consistent with the enhanced arachidonic acid metabolism pathway, its key enzyme COX2 was downregulated in the obese state but restored after exercise (Figure 1H). Similarly, exercise promoted the expression of β3-adrenergic receptor (ADRβ3) (Figure 1I) and thermogenic marker uncoupling protein 1 (UCP-1) (Figure 1J) in obese mice.

### 2.2. ADRβ3 Activation Promotes Lipolysis in Brown Adipocytes

**Figure 2:**
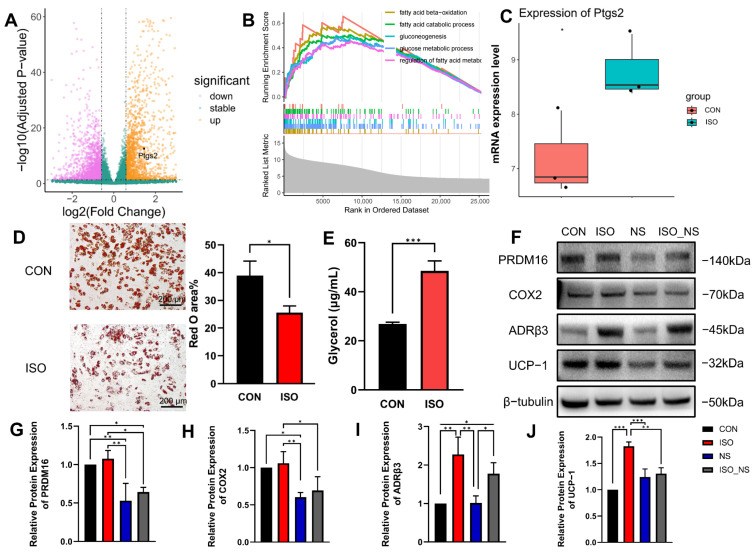
ADRβ3 activation promotes lipolysis in brown adipocytes. (**A**) Differentially expressed genes between the ISO-treated group and the control group (CON). (**B**) GSEA enrichment analysis shows significant upregulation of lipid metabolism pathways. (**C**) The COX2-encoding gene *Ptgs2* is significantly upregulated after ISO treatment. (**D**) Oil Red O staining and quantification analysis. Scale bar: 200 μm. ISO treatment reduces lipid droplet accumulation in brown adipocytes. (**E**) Glycerol quantification analysis indicates that ISO treatment promotes lipolysis. (**F**–**J**) Western blot analysis of the effects of ISO treatment and NS inhibition on the protein expression levels of PRDM16, COX2, ADRβ3, and UCP1. n = 3; CON: control group; ISO: isoproterenol-treated group; NS: NS-398-treated group, a COX2 inhibitor; ISO_NS: isoproterenol + NS-398 co-treatment group. * *p* < 0.05, ** *p* < 0.01, *** *p* < 0.001.

To investigate the metabolic effects of ADRβ3 activation in brown adipocytes and validate the previously identified target COX2, we performed RNA sequencing (RNA-seq) on brown adipocytes treated with isoproterenol (ISO, an ADRβ agonist) for 6 h and compared them to the control group. A total of 1356 upregulated genes and 1280 downregulated genes were identified (Figure 2A). Gene set enrichment analysis (GSEA) revealed that ISO treatment significantly upregulated multiple lipid metabolism-related pathways (Figure 2B), including that of prostaglandin-endoperoxide synthase 2 (Ptgs2), the gene encoding COX2, which showed a marked increase after ISO treatment (Figure 2C).

These findings were further validated by Oil Red O staining and glycerol content measurements. ISO treatment significantly reduced the lipid droplet area detected by Oil Red O staining (Figure 2D) and increased glycerol content. At the protein level, the COX2 inhibitor NS (NS-398) significantly suppressed the ISO-induced upregulation of the thermogenic marker UCP1 and the differentiation marker PRDM16 (Figure 2F,G,J).

In summary, ISO enhances brown adipocyte function by promoting lipid metabolism, with COX2 playing a key regulatory role in this process.

### 2.3. Inhibition of COX2 Impairs the Metabolic Enhancement Effect Induced by ADRβ3 Activation in Brown Adipocytes

**Figure 3:**
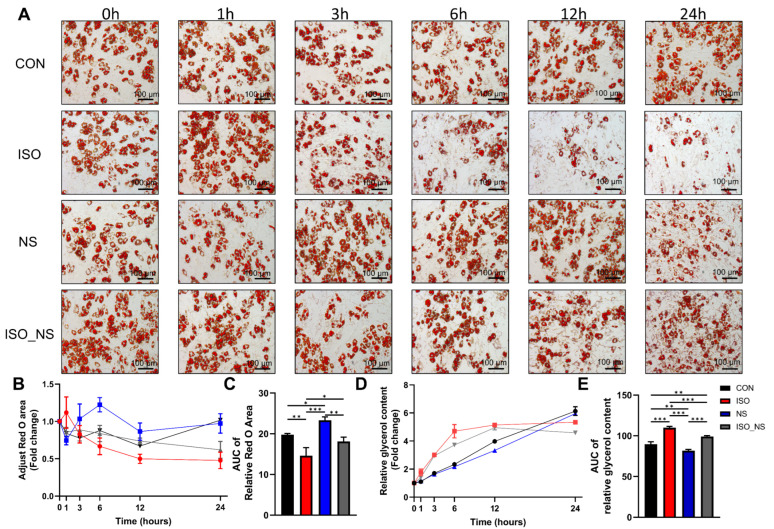
Inhibition of COX2 impairs the metabolic enhancement induced by ISO in brown adipocytes. (**A**) Representative Oil Red O staining images at six time points for the four treatment groups. Scale bar: 100 μm. (**B**) Oil Red O staining area for each treatment group at different time points. (**C**) Area under the curve (AUC) of Oil Red O staining, normalized to the 0 h baseline. (**D**) Glycerol quantification results showing lipolysis levels at different time points for each treatment group. (**E**) Area under the curve (AUC) of glycerol content, normalized to the 0 h baseline. n = 3; CON: control group; ISO: isoproterenol-treated group; NS: NS-398-treated group (COX2 inhibitor); ISO_NS: isoproterenol + NS-398 co-treatment group. * *p* < 0.05, ** *p* < 0.01, *** *p* < 0.001.

To further explore the temporal role of COX2 in ISO-induced brown adipocyte metabolism, we performed Oil Red O staining at multiple time points (0 h, 1 h, 3 h, 6 h, 12 h, and 24 h) to dynamically monitor the effect of COX2 inhibition on ISO-mediated lipolysis. The results showed that significant reduction in the lipid area appeared at 6 h in the ISO-treated group, whereas the inhibitory effect of the COX2 inhibitor NS became apparent starting at 12 h (Figure 3B). This finding was further validated by glycerol quantification analysis (Figure 3D). Additionally, the area under the curve (AUC) analysis of Oil Red O staining and glycerol content indicated that NS effectively inhibited overall lipid metabolism induced by ISO within 24 h (Figure 3C,E).

### 2.4. Time-Course Characteristics of RNA-Seq

**Figure 4:**
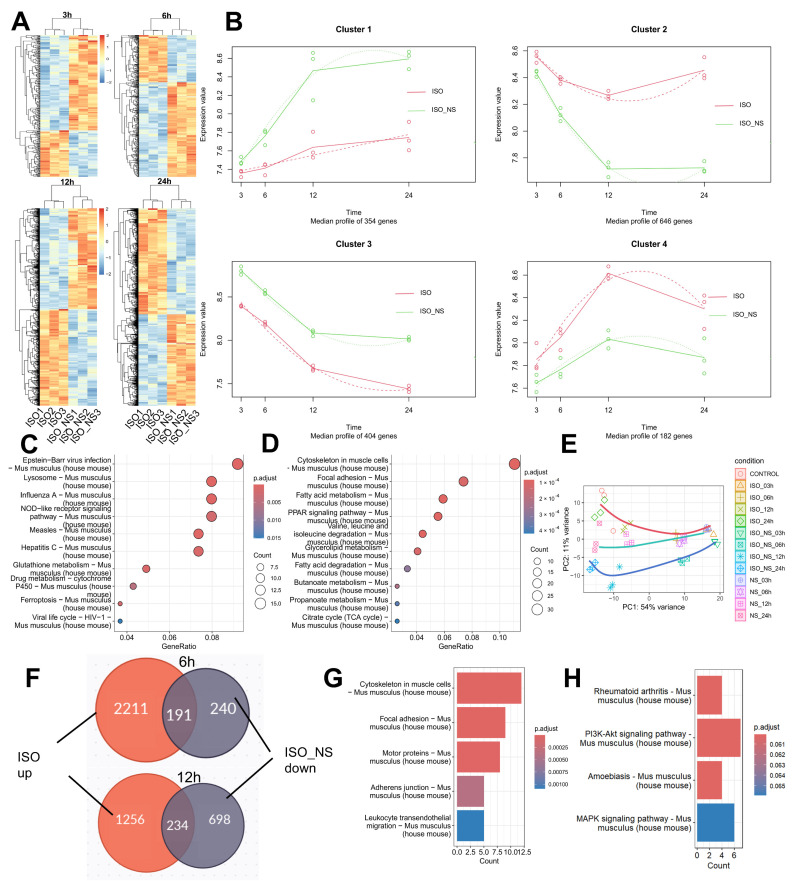
Time-course characteristics of RNA-Seq. (**A**) Heatmap of differentially expressed genes (DEGs) in brown adipocytes at 3 h, 6 h, 12 h, and 24 h under ISO and ISO_NS treatments. (**B**) Temporal expression patterns of four gene clusters. Solid line shows the fitted expression trend, and Dashed line shows the smoothed average change at each time point. (**C**) KEGG enrichment analysis of genes in cluster 1. (**D**) KEGG enrichment analysis of genes in cluster 2. (**E**) Principal component analysis (PCA) of ISO, ISO_NS, NS, and CON conditions. The lines in the figure represent the fitted trends of principal components over time for each group (**F**) Venn diagram showing common genes upregulated in ISO vs. CON and downregulated in ISO_NS vs. ISO. (**G**) KEGG enrichment analysis of common genes at 6 h. (**H**) KEGG enrichment analysis of common genes at 12 h. CON: control group; ISO: isoproterenol-treated group (ADRβ3 activation); NS: NS-398-treated group (COX2 inhibitor); ISO_NS: isoproterenol + NS-398 co-treated group.

To further investigate the time-dependent effects, we analyzed the differentially expressed genes (DEGs) in ISO_NS- and ISO-treated brown adipocytes at 3 h, 6 h, 12 h, and 24 h. The results showed that the number of DEGs gradually increased over time (Figure 4A). Next, we performed a detailed time-course analysis of DEG clusters using the maSigPro package and determined the optimal number of clusters (k = 4) based on the elbow method (Supplementary Figure S1).

The analysis revealed that genes in cluster 1 and cluster 2 exhibited the most significant changes over time, with ISO_NS treatment accelerating the decline in gene expression in these clusters (Figure 4B). The trend for cluster 1 indicated that compared to the ISO group, the ISO_NS group exhibited a more pronounced cellular stress response at multiple time points (Figure 4C). At 12 h, the expression of genes in cluster 2 reached its lowest point under ISO_NS treatment, whereas it remained high in the ISO group. GO enrichment analysis showed that these genes were primarily involved in lipid metabolism pathways (Figure 4D), consistent with the results in Section 2.2. Notably, the cytoskeleton pathway was most significantly enriched in the genes of cluster 2.

Principal component analysis (PCA) revealed differences between samples (Figure 4E). At 3 h, the differences between the ISO and/or NS groups and the CON were most pronounced. As time progressed, the differences between the treated groups and the CON gradually diminished, while the differences between the ISO and ISO_NS groups increased.

To clarify how NS inhibits ISO-induced metabolic activation in brown adipocytes, we selected genes that were upregulated under ISO treatment but downregulated at 6 h and 12 h in the ISO_NS-treated group (Figure 4F). KEGG enrichment analysis showed that at 6 h, these genes were significantly enriched in the cytoskeleton rearrangement and focal adhesion pathways (Figure 4G), while at 12 h, they were primarily enriched in the PI3K-Akt and MAPK signaling pathways (Figure 4H).

### 2.5. WGCNA and Machine Learning Identify Ywhah as a Key Feature Gene

**Figure 5:**
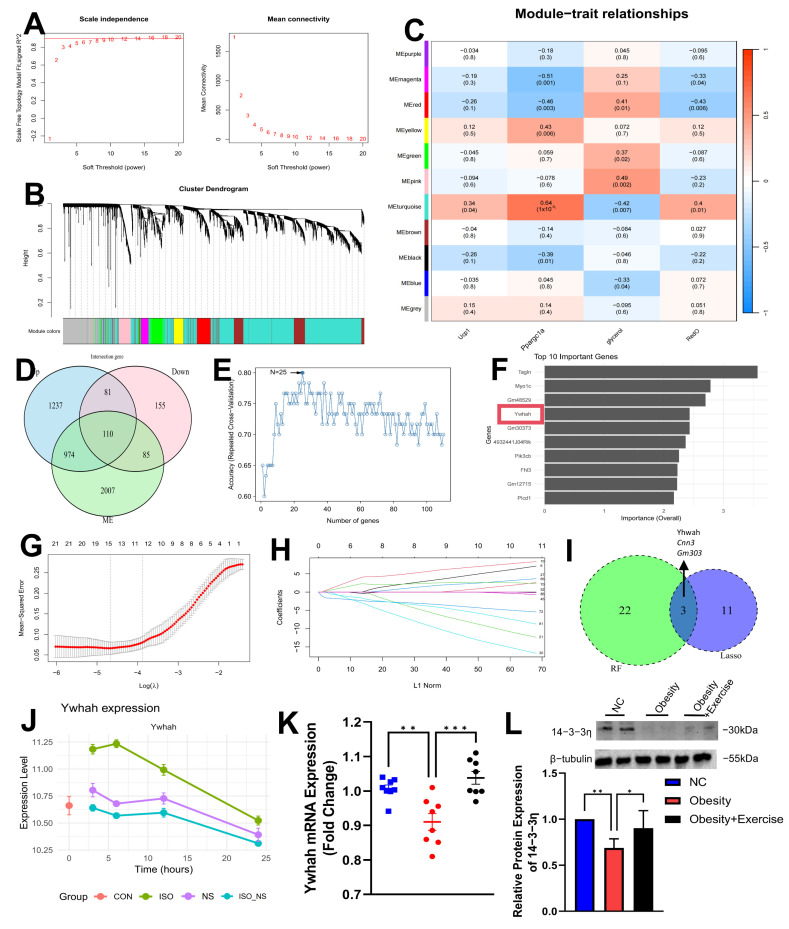
Identification of Ywhah as a key feature gene using WGCNA and machine learning methods. (**A**) Selection of the soft threshold parameter for constructing co-expression modules. (**B**) Identification of co-expressed gene modules. Different colors represent distinct gene co-expression modules identified by hierarchical clustering. (**C**) Heatmap of module–trait correlations. (**D**) Venn diagram showing genes upregulated in ISO vs. CON, downregulated in ISO_NS vs. ISO, and related to the identified modules. (**E**,**F**) Gene selection based on the Random Forest model. (**G**,**H**) Gene selection based on the Lasso regression model. (**I**) Venn diagram showing genes selected by both machine learning methods. (**J**) Expression trend of Ywhah across different groups in brown adipocytes. (**K**) Validation of Ywhah mRNA expression in brown adipose tissue of obese mice after 8 weeks of aerobic exercise, each n = 8. (**L**) Validation of 14-3-3η protein expression in brown adipose tissue of obese mice after 8 weeks of aerobic exercise, each n = 8. CON: control group; ISO: isoproterenol-treated group (ADRβ3 activation); NS: NS-398-treated group (COX2 inhibitor); ISO_NS: isoproterenol + NS-398 co-treated group. * *p* < 0.05, ** *p* < 0.01, *** *p* < 0.001.

To identify feature genes distinguishing ISO and ISO_NS treatments in brown adipocytes, we performed hierarchical clustering and constructed a weighted gene co-expression network (WGCNA) with a soft threshold of 9 (Figure 5A,B). Among the 11 identified gene modules, the MEturquoise module showed significant correlations with the expression of UCP1 and Ppargc1a, as well as the glycerol content and Oil Red O staining area (Figure 5C). In this module, we selected 110 genes that were upregulated in the ISO group and downregulated in the ISO_NS group (Figure 5D).

Next, we applied two machine learning algorithms, Random Forest (RF) and Lasso regression, to further refine these 110 genes. RF achieved the highest accuracy with a selection of 25 genes (Figure 5E,F), while Lasso regression identified 14 genes (Figure 5G,H). Venn diagram analysis revealed that Ywhah, Cnn3, and Gm30373 were selected by both methods (Figure 5I). Notably, Ywhah was identified as a key feature gene in the RF analysis. At multiple time points, Ywhah was significantly upregulated in the ISO group but downregulated in the NS group (Figure 5J).

To validate these findings, we analyzed the mRNA and protein expression of Ywhah by RT-qPCR and Western blot, which showed that, after 8 weeks of aerobic exercise, Ywhah was significantly upregulated in the BAT of obese mice (Figure 5K,L).

### 2.6. The 14-3-3η Protein Encoded by Ywhah Binding with Cytoskeletal Proteins

**Figure 6:**
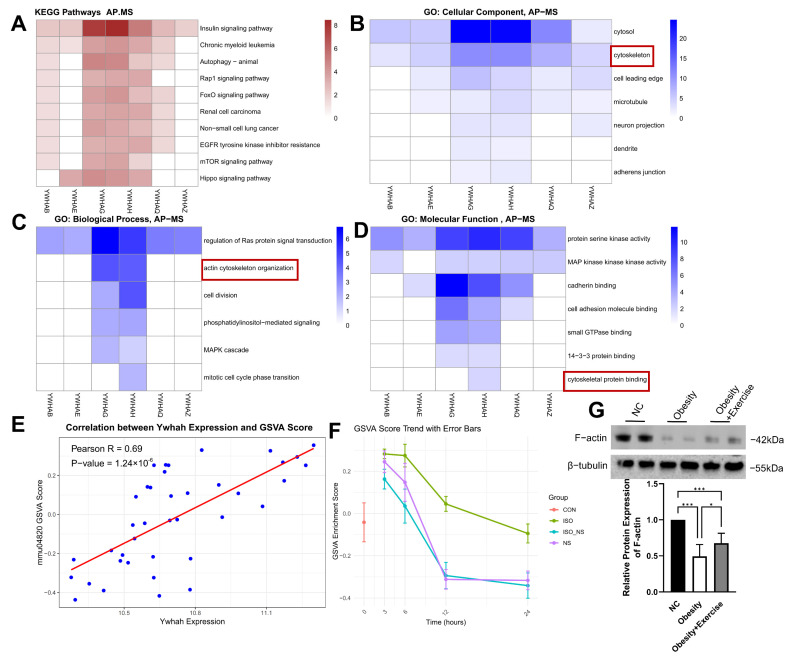
Ywhah expression and cytoskeleton interaction. (**A**) KEGG enrichment analysis of proteins interacting with the 14-3-3 family in the AP-MS experiment. (**B**–**D**) GO enrichment analysis. (**E**) Correlation score between Ywhah expression and cytoskeleton-related pathways. (**F**) Expression trends of the cytoskeleton pathway at different time points after treatment with ISO and/or NS in brown adipocytes. (**G**) Relative F-actin protein expression of mice (n = 8). AP-MS: affinity purification–mass spectrometry; CON: control group; ISO: isoproterenol-treated group (ADRβ3 activation); NS: NS-398-treated group (COX2 inhibitor); ISO_NS: isoproterenol + NS-398 co-treated group. * *p* < 0.05, *** *p* < 0.001.

Proteins of the 14-3-3 family function as molecular chaperones, but their roles remain underexplored. We analyzed the affinity purification–mass spectrometry (AP-MS) data provided by Dmitri et al. [22], and KEGG enrichment analysis revealed that proteins interacting with the 14-3-3 family are involved in several signaling pathways. Among them, YWHAG (14-3-3γ) and YWHAH (14-3-3η) exhibit broader interaction capabilities, particularly within the insulin signaling pathway (Figure 6A).

Interestingly, GO enrichment analysis revealed that YWHAH (14-3-3η) interacts significantly with cytoskeletal proteins (Figure 6B–D), and molecular function analysis within GO demonstrated that cytoskeletal protein binding is a unique feature of YWHAH (14-3-3η) (Figure 6D). Furthermore, our data showed a strong positive correlation between Ywhah expression and the GSVA score of cytoskeleton-related pathways in BAT (Figure 6E). Additionally, our study result showed that ISO treatment resulted in significantly higher activity of the cytoskeletal pathway compared to the NS treatment group in brown adipocytes (Figure 6F). Meanwhile, the cytoskeletal protein F-actin is also upregulated under the influence of exercise (Figure 6G). Given the close relationship between the cytoskeleton and the development and thermogenesis of BAT, these results suggest that ADRβ3/COX2 may enhance BAT metabolic function through Ywhah-mediated cytoskeletal remodeling.

## 3. Discussion

This study is the first to validate the role of the ADRβ3-COX2 pathway in brown adipocyte metabolism using the C3H10T1/2 cell line. Through WGCNA and machine learning analysis, we identified Ywhah as a potential key downstream molecule in this pathway. The protein encoded by Ywhah, 14-3-3η, may accelerate cytoskeletal remodeling in brown adipocytes by binding to cytoskeletal proteins, thereby promoting their development and enhancing metabolic function.

ISO (ADRβ3 agonist) promotes lipid metabolism, while NS-398 (COX2 inhibitor) suppresses this effect. Studies have shown that ADRβ3 receptors are abundant in BAT, and their short-term activation significantly enhances lipid metabolism [23], while long-term activation promotes BAT development [24]. In this study, both exercise and ISO treatment significantly enhanced lipolysis in brown adipocytes, while cells treated with both ISO and NS-398 exhibited larger lipid droplet areas and lower glycerol content. These results suggest that ISO-mediated lipolysis depends on COX2 activity. However, the role of COX2 in exercise remains controversial. In skeletal muscle, COX2 inhibition is associated with anti-inflammatory effects and reduced delayed-onset muscle soreness (DOMS) [25]. While this may be beneficial for high-intensity exercise, for exercise programs aimed at enhancing lipid metabolism, particularly those targeting fat reduction, activating COX2 expression may be the more suitable approach.

Through time-course RNA-seq analysis, we found significant temporal differences in lipid metabolism and stress-related pathways between ISO treatment alone and ISO combined with NS-398. We hypothesize that the high expression of stress-related genes may be linked to the disruption of phospholipid metabolic homeostasis caused by COX2 inhibition [26]. When cells are exposed to chemical or mechanical stimuli, membrane damage activates phospholipid metabolism, and COX2 serves as a key rate-limiting enzyme in this process [27]. In the absence of COX2 activity, disruptions in phospholipid metabolism may make cells more sensitive to stimuli such as ISO.

After ISO treatment, lipid metabolism in brown adipocytes remained active for up to 24 h but then gradually diminished, and the cellular state tended to return to baseline levels (CON). This phenomenon is reminiscent of the excess post-exercise oxygen consumption (EPOC), where the metabolic effects activated by exercise continue for a period after the stimulus ends [28]. Furthermore, because ISO is a catecholamine-based broad-spectrum ADRβ agonist with a short half-life, it is rapidly metabolized by enzymes after exerting its effect [29]. This explains the gradual decline in brown adipocyte responses over time following ISO treatment.

Although the beneficial effects of exercise on the body are widely recognized [30], the complex interactions between signaling pathways make selecting downstream targets a challenging task [31]. In this study, we employed WGCNA and two machine learning methods to perform dimensionality reduction on high-throughput sequencing data and identified Ywhah as a key downstream gene in the ADRβ3-COX2 pathway. Ywhah encodes the 14-3-3η protein, which primarily functions to bind phosphorylated proteins and regulate downstream pathways [32]. Recent studies have linked the 14-3-3ζ isoform to energy metabolism, showing that its overexpression exacerbates obesity and is closely associated with the development of visceral fat [33], while the deletion of 14-3-3ζ affects the expression of lipolysis-related genes in adipocytes [34]. Although these findings come from different types of adipose tissues and 14-3-3 isoforms, they also suggest that 14-3-3η may play an important role in BAT metabolism. Notably, while 14-3-3 family proteins generally share similar functions, different isoforms exhibit distinct expression patterns and functional characteristics [35]. This is reflected in the affinity purification–mass spectrometry (AP-MS) data provided by Dmitri et al., where all 14-3-3 isoforms bind to proteins from several pathways (e.g., insulin signaling, Figure 6A). However, the function of binding to cytoskeletal proteins is unique to Ywhah. Both sequencing data from BAT after exercise and in vitro data from the ADRβ3 activation of brown adipocytes show that the cytoskeletal pathway undergoes the most significant changes. This supports the hypothesis that exercise may improve brown adipocyte cytoskeletal and metabolic functions through the ADRβ3/COX2-Ywhah axis.

However, this study has some limitations. Although our experimental results and external datasets validate the potential role of Ywhah in improving lipid metabolism, whether directly targeting Ywhah in BAT can intervene in obesity remains to be further investigated.

**Figure 7:**
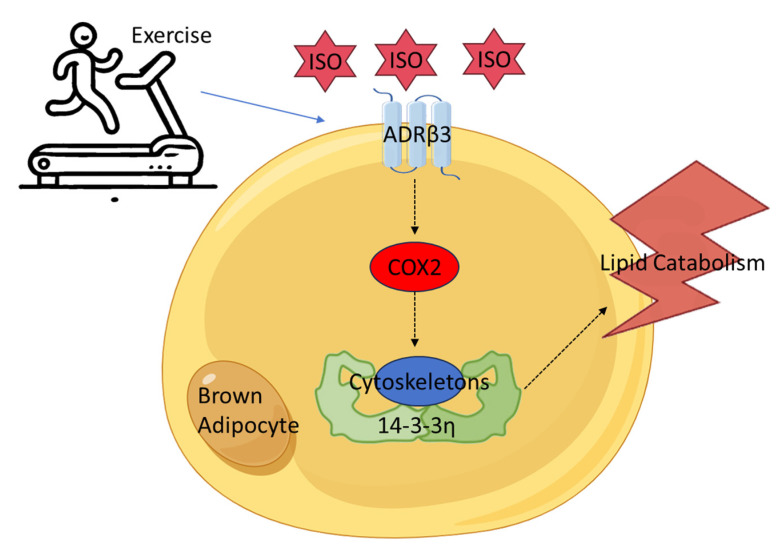
Hypothesis diagram.

In conclusion, this study preliminarily proposes the following scientific hypothesis: Exercise enhances the expression of Ywhah (14-3-3η) by activating the ADRβ3/COX2 pathway. 14-3-3η, by binding to key cytoskeletal proteins, maintains the dynamics and stability of the cytoskeleton, thereby promoting the development and metabolic function of BAT (Figure 7). This helps us uncover new mechanisms of metabolic regulation in BAT and deepens our understanding of how exercise improves obesity. It also provides scientific evidence for targeting Ywhah expression in BAT to combat obesity, offering potential benefits for individuals who are unable to exercise for various reasons.

## 4. Materials and Methods

### 4.1. Animals

A total of 24 male C57BL/6J mice (4 weeks old) were purchased from Vital River Laboratory Animal Technology Co., Ltd. (Beijing, China). The mice were housed in groups of 2–3 per cage in a controlled environment at 22 ± 2 °C and 40–70% humidity, with a 12 h light/12 h dark cycle. After 1 week of acclimatization, the mice were randomly divided into three groups (each n = 8): a normal diet control group (NC, 3.82 kcal/g, with 11.85% of the energy from fat, 23.07% from protein, and 65.08% from carbohydrates; the diet was provided by Beijing Keao Xieli Feed Co., Ltd. (Beijing, China)), an obesity group (4.46 kcal/g, with 40% of the energy from fat, 20% from protein, and 40% from carbohydrates; the diet was D12109C from Research Diets Inc. (New Brunswick, NJ, USA)), and an obesity exercise group. The obesity model was considered successfully established when the body weight of the obesity group was 20% higher than the average body weight of the NC group [21,22]. Mice in the obesity exercise group ran on a treadmill at a speed of 10 m/min for 1 h per day, 6 days per week, for 8 weeks. All animal experiments were approved in advance by the Sports Science Experiment Ethics Committee of Beijing Sport University on 29 December 2015 (Approval No. 2015040) and conducted in strict accordance with the ’Guidelines for the Management and Use of Laboratory Animals’ (Washington, DC, USA) issued by the relevant authorities.

### 4.2. Cell Culture

C3H10T1/2 cells (provided by the Cell Bank of the Chinese Academy of Sciences, Shanghai, China) were cultured under sterile conditions in DMEM/F12 medium containing 10% fetal bovine serum (Thermo Fisher Scientific, Waltham, MA, USA) at 37 °C with 5% CO₂. When the cells reached optimal confluence, they were transferred to differentiation medium (DMEM/F12 medium supplemented with 10% fetal bovine serum, 20 nM insulin, and 1 nM 3,3′,5-triiodo-L-thyronine) for 4 days, with medium changes every day. On day 4, the cells were transferred to brown adipocyte induction medium (differentiation medium supplemented with 2 μg/mL dexamethasone, 0.5 mM isobutylmethylxanthine, 0.125 mM indomethacin, and 1 μM rosiglitazone) for 2 days. Subsequently, the cells were returned to differentiation medium, and the medium was changed every other day. On day 10, fully differentiated brown adipocytes were divided into four groups: the control group (CON, treated with DMSO), the isoproterenol group (ISO, treated with 10 μM isoproterenol to activate β3-adrenergic receptors), the COX2 inhibiting group (NS, treated with 100 μM NS-398, a selective COX2 inhibitor), and the combined intervention group (ISO_NS, treated with both ISO and NS-398). Cell and culture supernatant samples were collected at 0, 3, 6, 12, and 24 h to observe the dynamic changes in adipocyte morphology and lipolysis in each group.

### 4.3. RNA-Seq

Total RNA was extracted from cell samples using TRIzol reagent. The RNA concentration and integrity were assessed using a NanoDrop2000 spectrophotometer (Thermo Fisher Scientific, Waltham, MA, USA) and Agilent 2100 Bioanalyzer (Agilent Technologies, Santa Clara, CA, USA), respectively. RNA libraries were constructed using the NEBNext Ultra RNA Library Prep Kit (New England Biolabs, Ipswich, MA, USA), following a workflow that included mRNA enrichment using magnetic beads, fragmentation, cDNA synthesis, end repair, A-tailing, adapter ligation, and PCR amplification. The library quality was evaluated before sequencing on an Illumina HiSeq platform using an Agilent 2100 Bioanalyzer and Qubit fluorometer (Thermo Fisher Scientific, Waltham, MA, USA). The sequencing was performed on the Illumina HiSeq platform, generating 150 bp paired-end reads, with approximately 8.28 Gb of data per sample.

Raw sequencing data were quality-controlled using SOAPnuke, with low-quality reads and adapter contamination removed. Clean reads were aligned to the reference genome (Mus musculus GRCm38.p5) using HISAT2, and gene expression levels were quantified using RSEM. Subsequent analyses were performed using R 4.4.0, including differential expression analysis, enrichment analysis, time-series analysis, weighted gene co-expression network analysis (WGCNA), and machine learning. Differential expression analysis was performed using the DESeq2 package, with significance defined as a fold change > 1.5 and an adjusted *p*-value < 0.05. Enrichment analyses included GSEA, GSVA, and KEGG pathway analysis. Time-series analysis was conducted using the maSigPro package, and WGCNA was performed using the WGCNA package. Machine learning models were implemented using the glmnet package for Lasso regression analysis, and the Random Forest model was constructed using the caret package. Visualization was performed using the ggplot2 package to ensure clear presentation and analysis of results.

### 4.4. Glycerol Quantification Assay

After aspirating the supernatant from the culture medium, glycerol concentration was measured using the Glycerol Assay Kit (Catalog No. 10011725, Cayman Chemical, Ann Arbor, MI, USA). Samples or standards were added to a 96-well plate, followed by the addition of glycerol assay reagent. After incubating for 15 min, the absorbance was measured at 562 nm. The glycerol concentration in the samples was calculated based on the standard curve.

### 4.5. Oil Red O Staining

After differentiation, cells were fixed with 4% paraformaldehyde for 5 min, followed by staining with Oil Red O solution (Catalog No. G1260, Solarbio, Beijing, China) for 15 min. After removing excess staining, cells were destained with 60% isopropanol and washed three times with distilled water. Finally, 500 μL of phosphate-buffered saline (PBS) was added as a mounting medium, and images were captured using an inverted microscope (DMI 4000 B, Leica Microsystems, Wetzlar, Germany). The percentage area of Oil Red O staining was quantitatively analyzed using ImageJ software (v 1.54).

### 4.6. Western Blotting

Protein extraction from brown adipose tissue or cells was performed using the Minute™ Total Protein Extraction Kit (Invent Biotechnologies, Plymouth, MN, USA). A total of 10 μg of protein was loaded per well and separated on a Bolt 4–12% Bis-Tris Plus gel (Invitrogen, Thermo Fisher Scientific, Waltham, MA, USA). Proteins were transferred to a nitrocellulose membrane using the iBlot 2 Gel Transfer Device and its associated iBlot 2 NC Regular Stack (Life Technologies, Carlsbad, CA, USA). The membrane was blocked with 5% bovine serum albumin (BSA) for 30 min, followed by incubation with the following primary and secondary antibodies: rabbit anti-ADRβ3 (AB94506, Abcam, Cambridge, UK), anti-COX2 (12375-1-AP, Protein Tech, Rosemont, IL, USA), anti-PRDM16 (AB106410, Abcam), anti-F-actin (AB Abcam), anti-UCP1 (AB23841, Abcam), rabbit anti-β-tubulin (BS4511R, BIOSS Inc., Woburn, MA, USA), and goat anti-rabbit secondary antibody (A9169, Sigma-Aldrich, Burlington, MA, USA).

Protein bands were visualized using the SuperSignal West Femto Enhanced Chemiluminescent Substrate (Thermo Fisher Scientific, Waltham, MA, USA), and imaging was performed using the ChemiDoc XRS+ System (Bio-Rad Laboratories, Hercules, CA, USA). Band intensity was quantified using Image Lab software (v6.1, Bio-Rad, Hercules, CA, USA) and normalized to β-tubulin as an internal reference.

### 4.7. RT-qPCR

Total RNA was extracted from brown adipose tissue (BAT) using the TaKaRa MiniBEST Universal RNA Extraction Kit, and complementary DNA (cDNA) was synthesized using the PrimeScript RT Master Mix (TaKaRa Bio Inc., Kusatsu, Shiga, Japan). RT-qPCR was performed using SYBR Premix Ex TaqII (TaKaRa Bio Inc., Kusatsu, Shiga, Japan) on the ABI 7500 Real-Time PCR System. A melting curve analysis was conducted to confirm the absence of any non-specific products. Then, 18S ribosomal RNA was used as the internal reference. Quantification was performed using the 2^−ΔΔCt^ method. The Ywhah primers were designed and validated for specificity using the NCBI online Primer Design Tool (Forward primer: TGGAAACTGACACAGCGACA; Reverse primer: GGCTGCTGAAAGCTCACAAG).

### 4.8. Statistical Analysis

All data are presented as the mean ± standard deviation (mean ± SD). Statistical analysis was performed using R version 4.4.0. Comparisons between two groups were conducted using Student’s *t*-test, while multiple group comparisons were performed using one-way analysis of variance (ANOVA) followed by Bonferroni’s post hoc test. Differences with a *p*-value less than 0.05 were considered statistically significant.

## Data Availability

The raw data will be provided upon request.

## Supplementary Materials

The following supporting information can be downloaded at https://www.mdpi.com/article/10.3390/ijms26072978/s1.

## Abbreviations

BAT	Brown Adipose Tissue
ADRβ3	Adrenergic Receptor Beta-3
COX2	Cyclooxygenase-2
ISO	Isoproterenol
WAT	White Adipose Tissue
Ywhah	14-3-3η (a protein encoded by the Ywhah gene)
WGCNA	Weighted Gene Co-expression Network Analysis
GSEA	Gene Set Enrichment Analysis
GSVA	Gene Set Variation Analysis
KEGG	Kyoto Encyclopedia of Genes and Genomes
