# Supplementary material for: Exercise Improves the Cytoskeletal and Metabolic Functions of Brown Adipocytes Through the ADRβ3/COX2-Ywhah Axis
Source: Int J Mol Sci. 2025 Mar 25;26(7):2978. doi: 10.3390/ijms26072978 (PMC11988500; doi:10.3390/ijms26072978)
Supplement: Supplementary file 1 [file ijms-26-02978-s001.zip › ijms-3516775-supplementary.pdf]

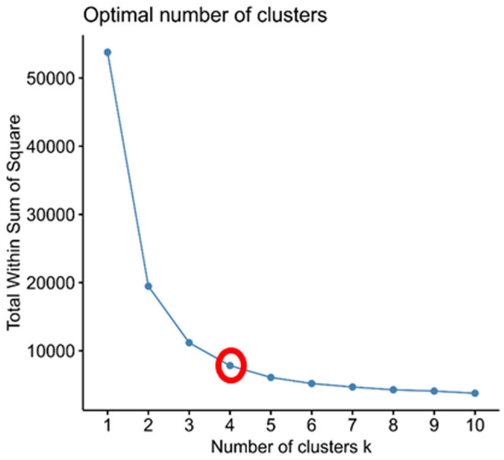

Supplementary Figure S1. Basis for the selection of the number of Masigpro clusters. Optimal cluster number determination method: Elbow method.
